# Thermal and Antioxidant Properties of Polysaccharides Sequentially Extracted from Mulberry Leaves (*Morus alba* L.)

**DOI:** 10.3390/molecules22122271

**Published:** 2017-12-20

**Authors:** Bu-Yan Liao, Dan-Ye Zhu, Kiran Thakur, Ling Li, Jian-Guo Zhang, Zhao-Jun Wei

**Affiliations:** 1 School of Food Science and Engineering, Hefei University of Technology, Hefei 230009, China; liaobuyan@afc.edu.cn (B.-Y.L.); danyezhu227@163.com (D.-Y.Z.); kumarikiran@hfut.edu.cn (K.T.); li97ling@163.com (L.L.); zhangjianguo@hfut.edu.cn (J.-G.Z.); 2 Department of Commerce, Anhui Finance & Trade Vocational College, Hefei 230601, China; 3 School of Life Science, Hefei Normal University, Hefei 230006, China

**Keywords:** polysaccharides, mulberry leaves, sequential extraction, thermal properties, antioxidant activities

## Abstract

Polysaccharides from natural plant products are gaining considerable attention due to their multi-faceted health effects, as well their functional applications in food production. We reported the sequential extraction of mulberry leaf polysaccharides (MLPs) with hot buffer (HBSS), chelating agent (CHSS), dilute alkali (DASS) and concentrated alkali (CASS), in order to obtain polysaccharide fractions. Monosaccharide analysis proved that galactose (27.07%) and arabinose (25.99%) were the major sugars in HBSS, whereas arabinose (30.55%) was the major sugar in CHSS, and glucose was the major sugar in DASS (24.96%) and CASS (27.51%). The molecular weights of the polysaccharide fractions were 7.812 × 10^3^ (HBSS), 3.279 × 10^3^ (CHSS), 6.912 × 10^3^ (DASS), and 1.408 × 10^3^ kDa (CASS). HBSS and CASS showed the largest peak temperature and the highest endothermic enthalpy, respectively. Different antioxidant assays showed that the MLPs possessed appreciable antioxidant activities in a dose-dependent manner. At 5 mg/mL, HBSS and DASS possessed the largest 2,2-diphenyl-1-picryl-hydrazyl-hydrate (DPPH) radical scavenging activity (96.82%). HBSS exhibited the highest reducing power, and DASS rendered the strongest ABTS radical scavenging activity (99.69%). CHSS performed the best hydroxyl radical scavenging activity (64.22%) and Fe^2+^-chelating ability (96.36%). Our results suggested that MLPs could be a promising source of natural antioxidants for use in the food, pharmaceutical, and cosmetic industries.

## 1. Introduction

Mulberry (*Morus alba* L.) is moraceous plant that holds a long history of cultivation, mainly in Asian countries. Its leaves and fruits are usually consumed as a food or food additive, and are also used in folklore medicine [1,2]. Mulberry leaves are recognized as the sole food source for silkworms, which are prosperous in the silk industry; they are also commonly used as anti-diabetic, hypolipidemic, anti-hypertensive, anti-atherosclerotic, and anti-convulsant agents [3,4,5,6,7,8,9,10]. Mulberry polysaccharides have been exploited as folk medicine to cure fever and liver diseases, as well as regulate blood pressure [11,12].

Polysaccharides are a heterogeneous group of carbohydrates that consist of monosaccharide units with glycosidic linkages [13] with diverse structures and compositions, which can influence various functional properties. It has been reported that polysaccharides have exhibited various biological activities such as anti-tumor, anti-inflammatory, anti-bacterial, and immunologic activity, as well as anti-mutagenic efficacy [14,15,16,17,18]. Moreover, polysaccharides also play an important role as radical scavengers to prevent oxidative damage [19]. Hence, it is necessary to explore these natural antioxidants in order to improvement the quality of food [20]. To the best of our knowledge, the structure and functions of polysaccharides possess significant diversity, which emerge in accordance with different extraction-processing methods [21]. Our previous reports indicated the sequential extraction of polysaccharides using four different solvents (hot buffer, chelating agent, dilute alkali, and concentrated alkali) from peony seed dreg [22], *Vaccinium bracteatum* Thunb. leaves [23], onion [24], and *Polygonatum cyrtonema* Hua [25]. Our results from these studies revealed important differences in chemical composition, monosaccharide composition, thermal characteristics, and antioxidant activities. To date, most of the reported extraction methods of mulberry leaf polysaccharides (MLPs) used traditional ultrasound and microwave-assisted techniques [26,27], and no report showed the sequential extraction of MLPs. Therefore, the objective of the present study was to investigate the physicochemical and antioxidant activities of the obtained polysaccharide factions from mulberry leaves. Herein, MLPs were sequentially extracted as soluble solids (SS) by hot buffer (HBSS), chelating agent (CHSS), dilute alkali (DASS), and concentrated alkali (CASS), followed by an investigation of their thermal and antioxidant properties. Polysaccharides were identified by UV absorption peak and Fourier transform infrared (FTIR) spectrum analysis. The molecular weights of the individual fractions were obtained by using high-performance liquid chromatography analysis, and monosaccharide composition was studied by gas chromatography analysis. Subsequently, the thermal characteristic was evaluated by differential scanning calorimetric (DSC) analysis, and antioxidant activities were evaluated by five different antioxidant assays in vitro. The obtained results manifested that MLPs could be used as essential candidates in the food industry due to their functional attributes, after a careful safety evaluation due to consumers’ health concerns.

## 2. Results 

### 2.1. UV Absorption Peak Detection

**Figure 1:**
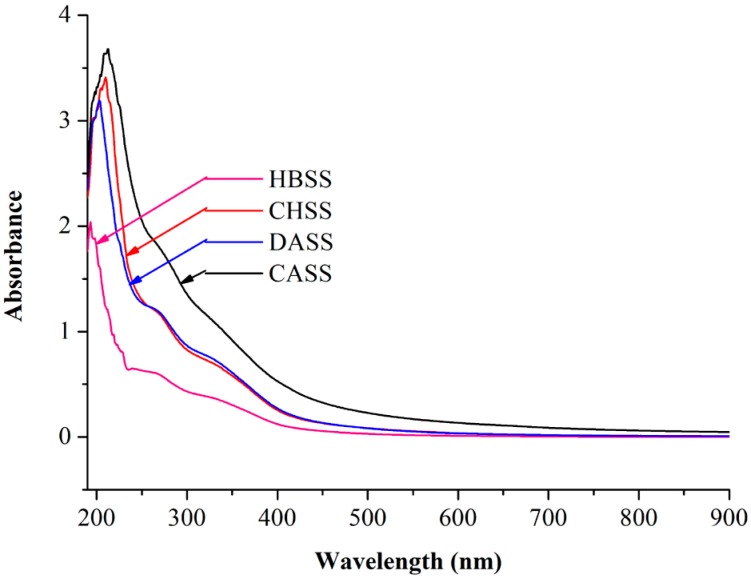
Ultraviolet scan of mulberry leaf polysaccharides (MLPs) over the range of 190–900 nm.

The complete ultraviolet scan for individual peaks of polysaccharides fractions is shown in Figure 1. The respective maximum absorption peaks of MLPs were located at 193 nm (HBSS), 210 nm (CHSS), 203 nm (DASS), and 212 nm (CASS).

### 2.2. Chemical Composition Analysis of MLPs

The yield, total sugar, and uronic acid contents of MLPs were determined and presented in Table 1. Among all of the factions, DASS had the largest yields (10.496 g/100 g AIS). CASS (85.46%) showed the highest total sugar contents, followed by HBSS (82.31%), CHSS (78.42%), and DASS (80.30%). Compared to all of the factions, the uronic acid content of CHSS (22.95%) was significantly (*p* < 0.05) higher than the others.

### 2.3. Determination of Molecular Weight and Monosaccharide Composition

**Figure 2:**
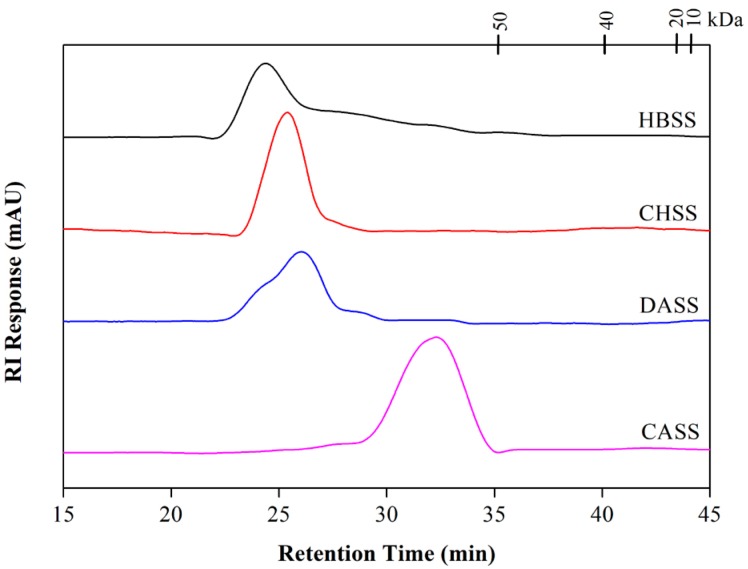
High-performance liquid chromatogram of MLPs.

According to high-performance liquid chromatography analysis, the molecular weights of MLPs are shown in Figure 2 and Table 2. All of the fractions displayed single peaks, which indicated their homogeneous natures. The molecular weights (MWs) of MLPs (HBSS, CHSS, DASS, and CASS) were 7.812 × 10^3^, 3.279 × 10^3^, 6.912 × 10^3^, and 1.408 × 10^3^ kDa, respectively.

The monosaccharide composition of MLPs is shown in Table 2. All of the four factions (HBSS, CHSS, DASS, and CASS) included rhamnose, arabinose, xylose, mannose, glucose, and galactose as major monosaccharide constituents. Our results showed that arabinose (25.99%) and galactose (27.07%) were the predominant monosaccharides of HBSS, whereas arabinose represented the CHSS. Moreover, the contents of glucose and galactose were highest in DASS (24.96% and 22.07%) and CASS (27.51% and 22.76%).

### 2.4. FTIR Spectrum Analysis of MLPs

**Figure 3:**
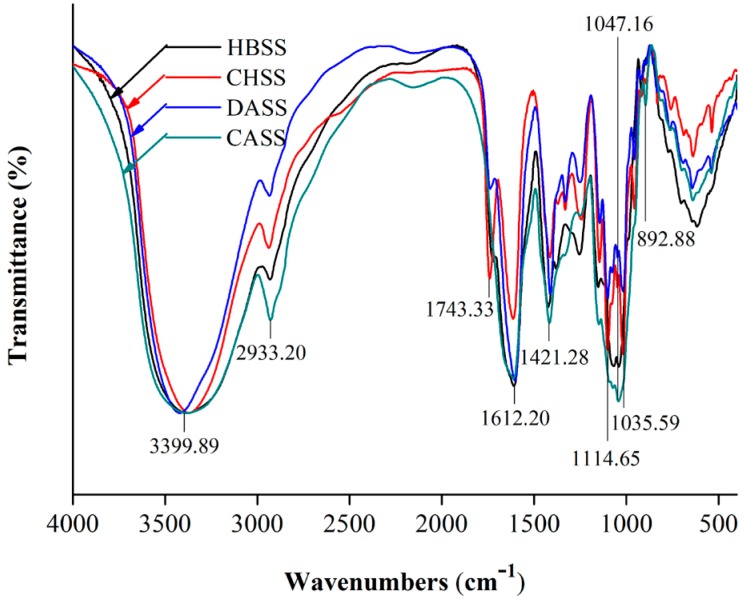
Fourier transform infrared (FTIR) spectrometric analysis of individual MLPs within the frequency range of 4000–400 cm^−1^.

FTIR spectrum was used to analyze the functional groups of polysaccharides, which are displayed in Figure 3 over the range of 4000–400 cm^−1^. The characteristic absorptions of 3600–3200 cm^−1^ and 1200–800 cm^−1^ indicated that the obtained fractions were of a polysaccharide nature. The broad and intense characteristic peaks around 3399.89 cm^−1^ represented an O–H stretching vibration [28]. The small band at 2933.20 cm^−1^ was attributed to the C–H absorption, which included CH, CH_2_, and CH_3_ stretching and bending vibrations, and may be due to the existing methyl ester group of galacturonic acid. The peaks observed at 1743.33 cm^−1^ were ester and carboxylic C=O vibrations [29]. Moreover, the signals at 1612.20 cm^−1^ and 1421.28 cm^−1^ represented that the MLPs had uronic acids that corresponded with the asymmetrical (C=O) and symmetrical (C–O) stretching vibrations [30]. The small peaks at 892.88 cm^−1^ represented the anomeric region due to the vibrational bands for α- and β-configuration being well separated [31]. The characteristic peaks at 1114.65 cm^−1^ for CHSS and DASS showed the presence of sulfate groups [24]. Moreover, the peaks at 1047.16 cm^−1^ corresponded to HBSS and CASS, while at 1035.59 cm^−1^ the peaks represented the CHSS and DASS, which were further attributed to glycosidic linkages [22,23].

### 2.5. Differential Scanning Calorimetric (DSC) Analysis

**Figure 4:**
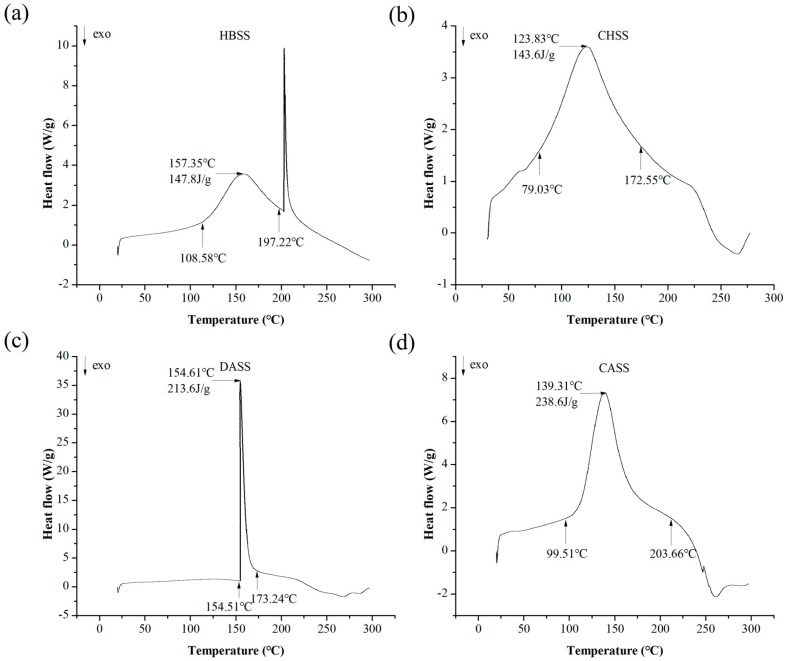
The differential scanning calorimetric (DSC) properties of MLPs. (**a**) HBSS; (**b**) CHSS; (**c**) DASS; (**d**) CASS.

Our differential scanning calorimetric (DSC) analysis of MLPs is summarized in Figure 4. The onset temperature (To) and conclusion temperature (Tc) ranged from 108.58 to 197.22 °C, 79.03 to 172.55 °C, 154.51 to 173.24 °C and 99.51 to 203.66 °C for HBSS, CHSS, DASS, and CASS, respectively. It may due to the change of the structure at the melting point. Four factions showed that the different peak temperatures in the range of 20–300 °C were 157.35 °C, 123.83 °C, 154.61 °C and 139.31 °C for HBSS, CHSS, DASS, and CASS, respectively. The endothermic enthalpy changes (ΔH) required to melt 1 g of four samples were 147.8, 143.6, 213.6 and 238.6, respectively. The endothermic enthalpy is generally required in order to calculate the required energy of the MLPs, which lead to disrupting the hydrogen bonds [32]. It may also owe to the thermal stability of MLPs.

### 2.6. Antioxidant Activities of MLPs

#### 2.6.1. DPPH Radical Scavenging Activity

**Figure 5:**
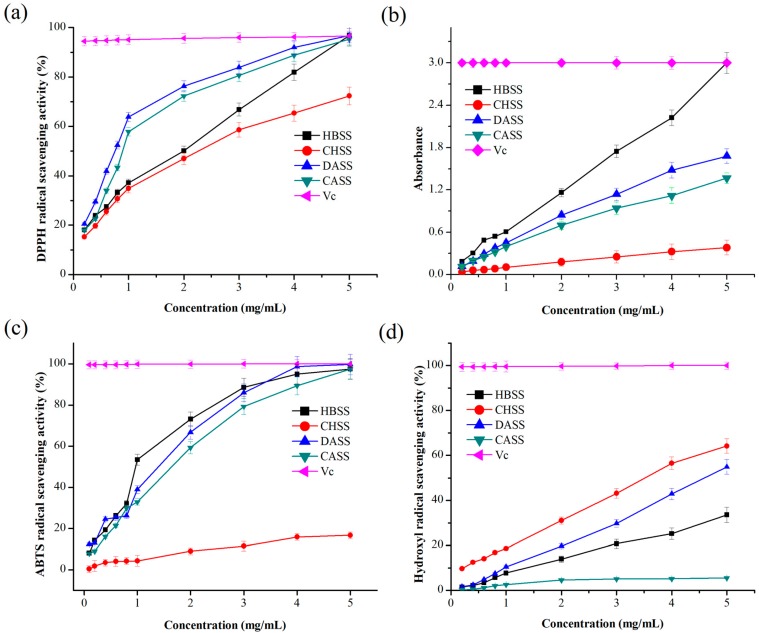
Antioxidant properties of MLPs at different concentrations. (**a**) 2,2-diphenyl-1-picryl-hydrazyl-hydrate (DPPH) radical scavenging activity; (**b**) Determination of reducing power; (**c**) 3-ethylbenzothiazoline-6-sulphonic acid (ABTS) radical scavenging activity; (**d**) Hydroxyl radical scavenging activity. Values were means ± SD of three replicates.

The 2,2-diphenyl-1-picryl-hydrazyl-hydrate (DPPH) radical is generally stable at room temperature, and the maximum absorbance is at 517 nm [28]. The DPPH radical scavenging activity is the ability to reduce the DPPH radical and oxidizing reaction, which depends on a structural conformation of the test sample, and changing the color of samples from purple to yellow [33]. The DPPH radical cation scavenging activity was improved with increasing polysaccharide concentration, as depicted in Figure 5a. It is apparent that CHSS (72.36%) displayed the lowest scavenging activity among all of the factions, whereas CASS had strong scavenging activity (95.31%), which was comparable to the activity of ascorbic acid/Vitamin C (Vc) (96.65%). Moreover, the scavenging activity of HBSS and DASS was 96.82%, which was stronger than Vc.

#### 2.6.2. Determination of Reducing Power

The reducing power assay was continually used to estimate the antioxidant activities by donating an electron or hydrogen atom, and it also represented which oxidation form of iron was changed to the ferrous form [34]. The results presented in Figure 5b show the reducing power of the MLPs. Reducing power was increased in a dose-dependent manner. At 5.0 mg/mL, the reducing power of the MLPs was in the following order: HBSS > DASS > CASS > CHSS. The result indicated that HBSS had a noticeable reducing power, which was the same as reported in Vc.

#### 2.6.3. ABTS Radical Scavenging Activity

The 3-ethylbenzothiazoline-6-sulphonic acid (ABTS) radical scavenging ability is widely applied to antioxidant activity. ABTS radicals after oxidation can change to a more stable form [35]. As shown in Figure 5c, the ABTS radical scavenging activity of the MLPs was correlated with increasing concentrations. The scavenging activity of CHSS was extremely weak at the largest concentration (16.62%), which showed almost no scavenging activity. Nevertheless, HBSS (97.49%), DASS (99.69%), and CASS (97.41%) displayed stronger scavenging activity, which was almost comparable to Vc (99.99%).

#### 2.6.4. Hydroxyl Radical Scavenging Activity

As per the previous report, the hydroxyl radical is produced under in vivo conditions from water or H_2_O_2_ [33]. The hydroxyl radical can easily cross cell membranes, react with biomolecules, and then cause cell death. Hence, removing the hydroxyl radical is important in cell or food systems [36]. The antioxidant activities of the hydroxyl radical in all of the samples are demonstrated in Figure 5d, which were observed to be in a dose-dependent manner. The scavenging power of MLPs was relatively weak, and the strongest ability was observed in CHSS (64.22%) compared with the other fractions at a concentration of 5.0 mg/mL.

#### 2.6.5. Iron (Fe^2+^) Chelating Activity

**Figure 6:**
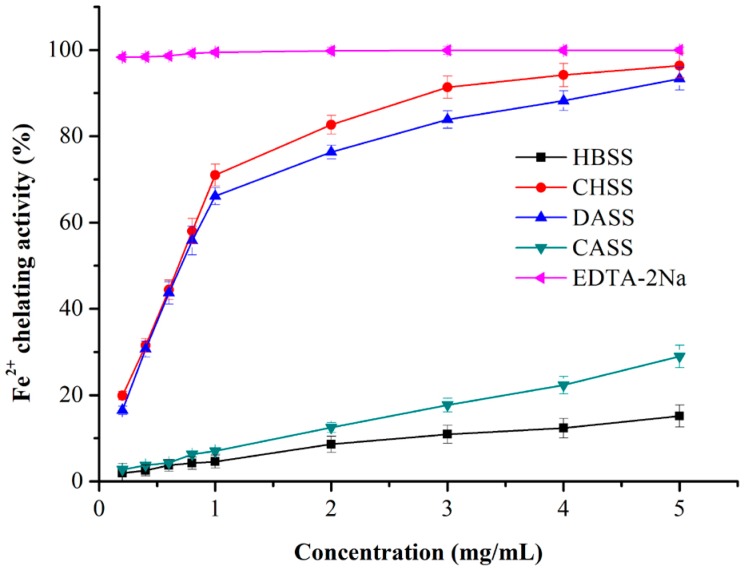
Iron (Fe^2+^)-chelating activity of MLPs at different concentrations.

It has been reported that ferrous ions are the most effective pro-oxidants in the food system [37]. The Fe^2+^-chelating ability of MLPs increased in a concentration-dependent manner, as shown in Figure 6. CHSS and DASS revealed a strong chelating ability that was comparable to EDTA-2Na at the largest concentration (5 mg/mL). However, HBSS and CASS displayed much lower activity. Moreover, the order of Fe^2+^-chelating activity was as followed: EDTA-2Na > CHSS > DASS > CASS > HBSS.

## 3. Materials and Methods

### 3.1. Materials and Chemicals

Mulberry leaves (*Morus alba* L.) were provided by the Anhui Academy of Agricultural Sciences (Anhui, China). They were dried, ground, further sieved (60-mesh size), and stored in a desiccator at room temperature for polysaccharides extraction. All of the chemicals used in the present study were of analytical grade.

### 3.2. Sequential Extraction of MLPs

**Figure 7:**
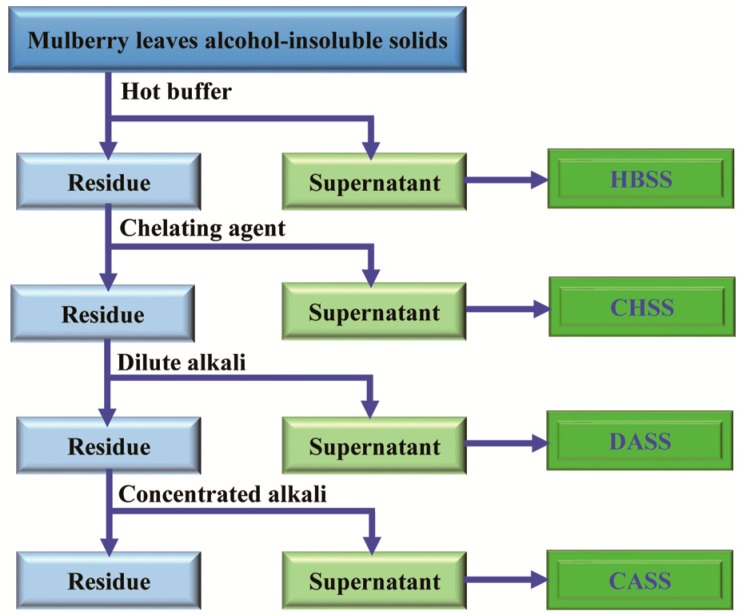
Flow diagram of the extraction procedure for different polysaccharide fractions.

Polysaccharides from mulberry leaves were extracted according to the method of Sengkhamparn et al. [38] with few modifications (Figure 7). The mulberry leaves powder was homogenized thrice with 70% (*v*/*v*) aqueous ethanol at room temperature and filtrated; then, the resultant insoluble residues were washed with three volumes of chloroform/methanol (1/1, *v*/*v*) for 45 min to eliminate the low molecular weight compounds. Subsequently, the insoluble residues obtained after filtration were washed by the addition of fourfold volume of acetone, and air dried to obtain alcohol-insoluble solids (AIS). Mulberry leaf AIS (20 g) was sequentially extracted with four different solvents, which were hot buffer, chelating agent, dilute alkali, and concentrated alkali, respectively. Briefly, the mulberry leaf AIS (20 g) was exposed to respective solvents such as 0.05 M sodium acetate buffer for 1 h (pH 5.2, 70 °C), 0.05 M EDTA and 0.05 M ammonium oxalate in 0.05 M sodium acetate buffer for 1 h (pH 5.2, 70 °C), 0.05 M sodium hydroxide and 20 mM NaBH_4_ (4 °C) for 1 min), and 6 M sodium hydroxide and 20 mM NaBH_4_ (4 °C) for 2 h, and the obtained crude polysaccharides were named HBSS, CHSS, DASS, and CASS, respectively. The obtained extracts were centrifuged at 7155 g for 25 min individually, and the resultant supernatants were dialyzed at 4 °C with a dialysis bag (molecular weight cut-off 8000 Da) in distilled water for two days. After dialysis was over, the remaining products were freeze-dried to obtain MLPs.

### 3.3. Purification of Crude Mulberry Leaf Polysaccharides

The lyophilized MLPs were dissolved in distilled water and purified sequentially by chromatography comprising of DEAE Cellulose-52 (Sigma-Aldrich, St. Louis, MO, USA), and the fractions were eluted successively with 0, 0.1, 0.2, and 0.3 mol/L NaCl solutions at a flow rate of 1 mL/min. Each fraction with 5 mL of eluate was collected and determined by the phenol-sulfuric acid method. Four purified MLPs were successively concentrated, dialyzed, and freeze-dried for further study. After extraction, the obtained fractions were subjected to purification followed by rotary evaporation for a few hours to ensure that the resulting polysaccharides were free from all kinds of chemicals used during extracting procedures.

### 3.4. UV Absorption Peak Detection

MLPs were dissolved in distilled water to obtain a desired concentration [35]. The UV absorption spectra (RT600, Guangdong, Shenzhen, China) of the MLPs were recorded (ranging from 190 nm to 900 nm).

### 3.5. Chemical Composition Analysis of MLPs

The yield of MLPs was determined by weighing the dried samples of each fraction. Total sugar content was examined by phenol-sulfuric acid colorimetric methods using d-glucose as the standard. Subsequently, the uronic acid content was calculated according to the *m*-hydroxydiphenyl method using galacturonic acid as the standard. All of the measurements were performed in triplicate [31].

### 3.6. Homogeneity and Molecular Weight Determination

The determination of the molecular weights of purified polysaccharides was performed using a high-performance liquid chromatography (Waters E2695) (Model LC 1100, Agilent Co., Santa Clara, CA, USA) [31], which was equipped with an Ultrahydrogel™ Linear (1.8 mm × 300 mm i.d. × 2) gel-filtration column (Model G4000 PWXL, Tosoh Co., Minato-Ku, Japan), and the column temperature was 25 °C. Ultrapure water was used as the flow phase at a flow rate of 0.6 mL/min, and the injection volume of the samples was 20 μL.

The molecular weight was determined by the calibration curve obtained from standard dextrans of different molecular weights (10, 20, 400 and 500 kDa).

### 3.7. Monosaccharide Composition of MLPs

The monosaccharide composition was determined by gas chromatography (GC) analysis (model-8700, PerkinElme, Waltham, MD, USA) [39]. For this, trifluoroacetic acid (TFA) (4 mL 2 mol/L) (St. Louis, MO, USA) was added to 4 mg lyophilized MLPs. The mixture was hydrolyzed for 4 h at 120 °C, then the hydrolysate was evaporated to remove the residual TFA. Subsequently, the solutions were added to 30 mg of NaBH_4_, kept for 4 h, and neutralized with 25% CH_3_COOH. The resultant was mixed with CH_3_OH using a rotary vacuum evaporator to remove water and CH_3_COOH. After drying at 120 °C in an oven for 20 min, the residues were added to 3 mL acetic anhydride and 3 mL pyridine at 100 °C for 1 h. Finally, the residues were added to 3 mL water and 1 mL of chloroform to remove the water phase, and the GC analysis was performed.

### 3.8. Infrared Spectrum Analysis of MLPs

The FTIR spectrum of MLPs was performed using a Thermo Nicolet 67 FTIR spectrometer equipped with a deuterated triglycine sulphate (DTGS) detector with a resolution of 0.09 cm^−1^ (Thermo Nicolet Corporation, Waltham, MD, USA) [22]. The polysaccharide samples were grounded with dried potassium bromide (KBr) powder and pressed into a 1.0-mm thick pellet. Spectrum analysis was recorded in the frequency range of 4000–400 cm^−1^.

### 3.9. Differential Scanning Calorimetric (DSC) Analysis

Differential scanning calorimetry was used for the determination of the thermal properties of polysaccharides, according to the previous method, with slight modifications [22,23,24]. Approximately 4 mg of MLPs was placed in an aluminum pot, sealed, and kept at a stable heating rate of 10 °C/min in the range of 20–300 °C under nitrogen atmosphere (50 mL/min).

### 3.10. Antioxidant Activities of MLPs

#### 3.10.1. DPPH Radical Scavenging Activity

The DPPH radical scavenging activity was assessed according to the previously described method with minor modifications [28]. MLPs were pre-diluted to a concentration range of 0.2, 0.4, 0.6, 0.8, 1.0, 2.0, 3.0, 4.0 and 5.0 mg/mL prior to analysis. Briefly, 2.0 mL of the sample solutions were added to a freshly prepared DPPH ethanol solution (2.5 mL, 0.1 mM), and incubated for 40 min in the dark at room temperature. The scavenging activity was determined at 517 nm. Ethanol and ascorbic acid (Vc) were used as blank and positive controls, respectively. The DPPH radical scavenging activity was calculated as follows:

DPPH radical scavenging activity (%) = (A_0_ − A_1_ + A_2_)/A_0_ × 100 (1)

A_0_ was the absorbance value of ultrapure water instead of samples, A_1_ was the absorbance value of the solution with different concentrations of samples, and A_2_ was the absorbance value of the samples with ultrapure water.

#### 3.10.2. Determination of Reducing Power

The antioxidant activity was evaluated by testing the reducing power according to the previously reported method, with some modifications [34]. Briefly, the different concentrations of samples (1 mL) were mixed with 1 mL of 0.2 M phosphate buffer (pH 6.6) and 1 mL of 1% potassium ferricyanide. After incubation at 50 °C for 20 min, the reaction of mixture was stopped by adding 1 mL of 10% trichloroacetic acid (TCA), and then centrifuged at 1914× *g* for 5 min. The supernatant (1 mL) was mixed with 1 mL of ultrapure water and 0.5 mL of ferric chloride (0.1%), and further incubated for 10 min at room temperature. The absorbance was measured at 700 nm, and Vc was used as a positive control.

#### 3.10.3. ABTS Radical Scavenging Activity

The ABTS radical scavenging activity was performed using the previously reported method with a few modifications [35]. ABTS solution (7 mM) mixed with potassium persulfate (4.5 mM) was allowed to react in the dark at room temperature for 12–16 h. The ABTS radical cation solution was diluted in ultrapure water to give an initial absorbance of 0.7 at 734 nm before use. Then, 2 mL of diluted ABTS was added to different concentrations of samples (0.5 mL) and incubated for 5 min at room temperature. The absorbance at 734 nm was measured. Vc was used as a standard. The ABTS radical scavenging activity was calculated by the following equation:

ABTS radical scavenging activity (%) = (A_0_ − A_1_ + A_2_)/A_0_ × 100 (2)

A_0_ represented the absorbance value of the ABTS solution without samples, A_1_ represented the absorbance value of the test samples, and A_2_ was the absorbance value of samples without an ABTS solution.

#### 3.10.4. Hydroxyl Radical Scavenging Activity

The hydroxyl radical scavenging activity was evaluated using the modified method mentioned, with some modifications [33]. Samples were dissolved in ultrapure water to obtain the desired concentration range of 0.2, 0.4, 0.6, 0.8, 1.0, 2.0, 3.0, 4.0 and 5.0 mg/mL. The reaction mixture containing 1 mL of sample solutions, 1 mL of ferrous sulfate (8 mM), 1 mL of salicylic acid (8 mM), and 0.5 mL of H_2_O_2_ (0.1%) was incubated at 37 °C for 30 min and measured at 510 nm for each fraction. Vc was used as a reference standard. The scavenging activity of the hydroxyl radical was calculated using the following formula:

Hydroxyl radical scavenging activity (%) = (A_0_ − A_1_ + A_2_)/A_0_ × 100 (3)

A_0_ corresponded to the blank control, A_1_ represented the absorbance value of the sample and the positive control, and A_2_ was the absorbance value of the sample with 3.0 mL ultrapure water.

#### 3.10.5. Iron (Fe^2+^)-Chelating Activity

The Fe^2+^-chelating ability was determined according to the previous report, with minor modifications [37]. First, 1 mL of MLPs solution was mixed with 0.1 mL of ferrous chloride solution (2.0 mM), 0.2 mL of ferrozine solution (5 mM), and 2.5 mL of ultrapure water. Subsequently, the resulting mixtures were incubated for 10 min at room temperature, and the absorbance was measured at 562 nm. EDTA-2Na was used as a positive control. The Fe^2+^-chelating activity was given by the formula below:

Fe^2+^-chelating activity (%) = (A_0_ − A_1_ + A_2_)/A_0_ × 100 (4)

A_0_ was the absorbance value of the blank control, A_1_ was the absorbance value of the sample solutions at different concentrations, and A_2_ was the absorbance value of the sample with ultrapure water.

### 3.11. Statistical Analysis

Data were expressed as the mean values ± standard deviation (SD) in triplicate. The data analysis was performed using a statistical software program (SPSS for Windows version 16) (SAS Institute Inc., Cary, NC, USA) and analysis of variance (ANOVA) (SAS Institute Inc., Cary, NC, USA) (which was followed by Duncan′s multiple comparison (significance level *p* < 0.05).

## 4. Discussion

Previously, our research group studied the thermal and rheology characteristics of plant-derived polysaccharides and their functional properties [14,23,24,25,40], but the study of the thermal and rheological characteristics of mulberry polysaccharides is still in its infancy. We reported the sequential extraction of MLPs with different solvents such as hot buffer, chelating agent, dilute alkali, and concentrated alkali, which led to considerable differences in yield and chemical composition. Subsequently, these differences led to unique thermal and antioxidant properties of respective polysaccharides fractions. The different UV absorption peaks of four fractions may be due to the different structural composition of the polysaccharides. Our data expressed that MLPs were homogenous polysaccharides and possessed a slight content of protein or nucleic acid, because of the small peaks at 250–280 nm [35]. Among all of the factions, DASS had the largest yields (10.496 g/100 g AIS), which were lower than our previous reports of onion polysaccharides (52.65 mg/g AIS) [24]. Our results showed the dissimilarity in the sugar composition in all of the fractions, which may due to the special solubilization patterns of polysaccharides in different solvents with different extraction procedures and conditions of purification [31]. Further, the uronic content of the CHSS fraction can be related to its moisturizing ability, which makes it a suitable candidate to be used in cosmetics [24]. Moreover, our results showed the diverse monosaccharide composition in all four fractions. The results may be due to the different materials and purifying methods used. To support this statement, from our previous studies related to polysaccharides extraction and their biological properties, we could demonstrate that extraction methods largely impact the chemical composition and physiological properties of the obtained factions. The composition, molecular weights, and yield of obtained fractions vary significantly with the respective extraction procedures. Various buffer treatments also resulted in the biological activities of obtained polysaccharides changing, as reported previously [14,23,24,25,39,40]. Moreover, uronic acid content might be related to the higher molecular weights obtained. Further, the latter are likely to be overestimated when using dextran as a standard, which may be a limitation of the present study. These different characteristics may affect the biological activities of individual polysaccharides. The previous reports have also suggested that the biological potential of polysaccharides was largely dependent on the contents of mannose and rhamnose [39]. FTIR spectrum analysis demonstrated the functional groups of obtained polysaccharides at particular wavelength ranges. In this study, CASS had the largest temperature stability, which was in accordance with the results of polysaccharides from peony seed dreg [22], but different from the polysaccharides from *V. bracteatum* Thunb. leaves [23] and onion [24]. Our results indicated that CASS is better to be used as a supplement in hot processed foods. Different antioxidant assays showed that MLPs possessed appreciable antioxidant activities in a dose-dependent manner. In our study, we observed large differences between the DPPH scavenging abilities of the four fractions. The previous studies have shown that the scavenging activity of polysaccharide fractions may be affected by their structure, molecular weight, and monosaccharide component [41,42]. Similarly, the different hydroxyl radical scavenging activity indicated that the difference might be attributed to some structural or chemical changes after extracted processing [36]. In the case of iron-chelating ability, the results were similar to our previous study, which may due to CHSS and DASS having some functional groups, such as -OH, -SH, -COOH, and C=O [43,44]. Overall, our results suggested that MLPs could be promising sources of natural antioxidants for use in the food, pharmaceutical and cosmetic industries. To summarize, our data demonstrated that extraction solvents have impacted antioxidant activities to a great extent. The fraction CHSS possessed the largest hydroxyl radical scavenging activity and Fe^2+^ chelating, whereas HBSS showed the strongest DPPH radical scavenging activity and reducing power. DASS possessed the best DPPH radical scavenging activity and ABTS radical scavenging activity. The different fractions can be selectively used for the particular function for each antioxidant attribute, as shown by the different assays used in this study. Hence, it is essential to obtain polysaccharides from natural sources and characterize their functional properties.

## 5. Conclusions

The aim of this work was to characterize the mulberry polysaccharides, namely HBSS, CHSS, DASS, and CASS, which were sequentially extracted with four kinds of solvents (hot buffer, chelating agent, dilute alkali, and concentrated alkali), respectively. MLPs displayed several differences in the physicochemical and antioxidant activities. Four polysaccharide fractions exhibited characteristic absorptions spectra and sugar composition, as well as distinct molecular weights upon treatment with different solvents. Besides the sugar content, nucleic acid, and protein contents may also be responsible for antioxidant properties to some extent, which warrants further investigation in the future. The thermal properties indicated the suitability of CASS as a supplement in hot processed foods. Furthermore, MLPs displayed diverse antioxidant activities. For example, CHSS possessed the largest hydroxyl radical scavenging and Fe^2+^-chelating activities, while HBSS showed the strongest DPPH radical scavenging activity and reducing power. DASS possessed the best DPPH and ABTS radical scavenging activities. The different antioxidant assays performed indicated that MLPs might be considered natural antioxidants in different food and medical industries according to the unique characteristics of each fraction after safety evaluations for public health concerns. Therefore, further in-depth studies are warranted in order to understand the underlying mechanisms and carry the importance of the obtained fractions further in the future.

## Figures and Tables

**Table 1:** Yield, sugar composition, and uronic acid content of MLPs. Values were expressed as mean ± SD of three replicates, with different letters indicating significant differences at level of *p* < 0.05. HBSS: Hot buffer; CHSS: Chelating agent; DASS: Dilute alkali; CASS: Concentrated alkali.

Samples	Yield (g/100 g AIS)	Total Sugar Content (%)	Uronic Acid (%)
HBSS	9.4 ± 0.4 ^b^	82.3 ± 3.5 ^a,b^	12.4 ± 0.5 ^d^
CHSS	5.2 ± 0.2 ^c^	78.4 ± 3.1 ^b^	22.9 ± 0.8 ^a^
DASS	10.5 ± 0.5 ^a^	80.3 ± 3.2 ^a,b^	14.7 ± 0.6 ^c^
CASS	5.7 ± 0.3 ^c^	85.5 ± 3.5 ^a^	20.8 ± 0.9 ^b^

Values of a–d are significantly different with different fractions methods at significance level of *p* < 0.05.

**Table 2:** The molecular weight and monosaccharide compositions of polysaccharides fractions. Rha: Rhamnose; Ara: Arabinose; Xyl: Xylos; Man: Mannose; Glu: Glucose; Gal: Galactose.

Samples	Molecular Weight (kDa)	Sugar Components (%)					
		Rha	Ara	Xyl	Man	Glu	Gal
HBSS	7.812 × 10^3^	21.18	25.99	2.22	4.96	18.58	27.07
CHSS	3.279 × 10^3^	26.11	30.55	2.76	2.11	17.96	20.51
DASS	6.912 × 10^3^	11.96	20.42	17.89	2.70	24.96	22.07
CASS	1.408 × 10^3^	13.69	18.92	14.18	2.94	27.51	22.76

## Supplementary Materials

Supplementary File 1
