# Supplementary material for: Thermal and Antioxidant Properties of Polysaccharides Sequentially Extracted from Mulberry Leaves (Morus alba L.)
Source: Molecules. 2017 Dec 20;22(12):2271. doi: 10.3390/molecules22122271 (PMC6149993; doi:10.3390/molecules22122271)
Supplement: Supplementary File 1 [file molecules-22-02271-s001.pdf]

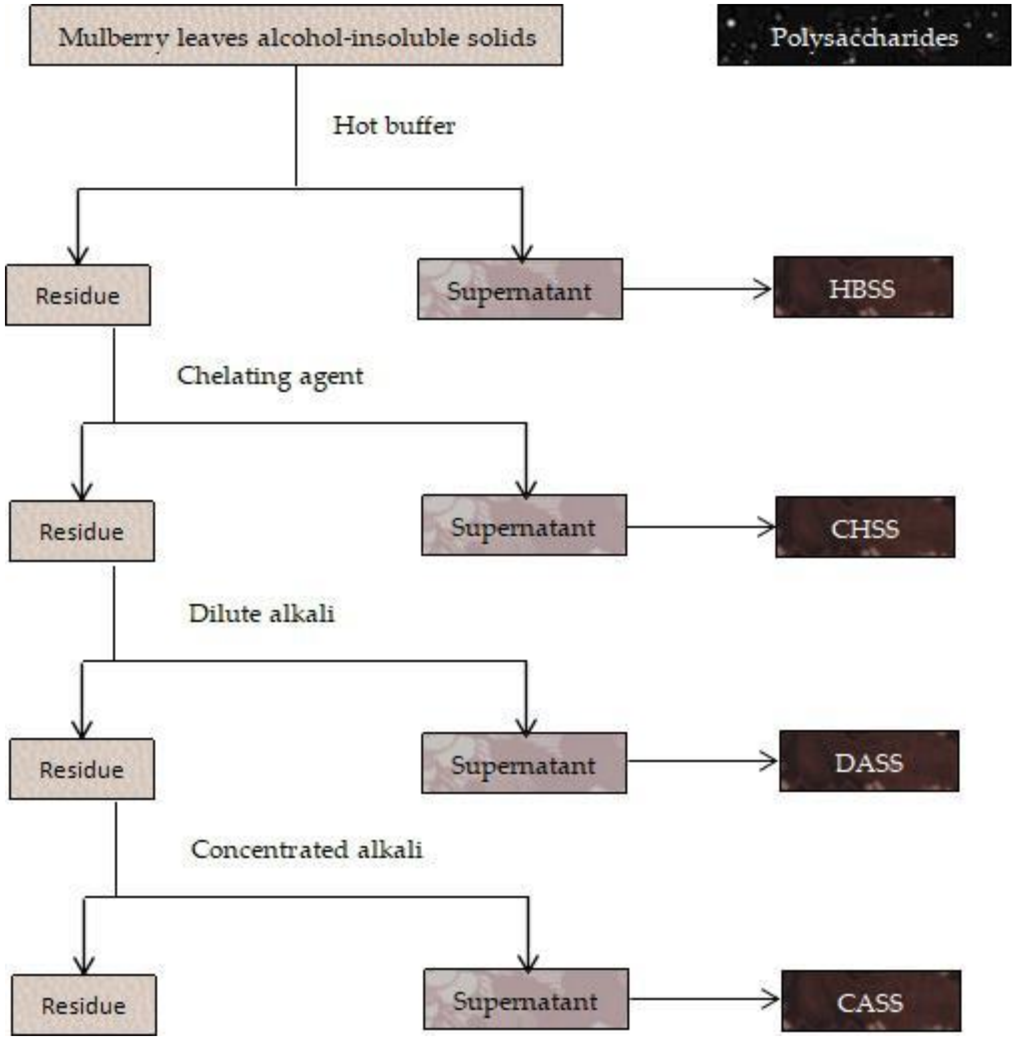

Supplement Figure 1: Flow diagram of extraction procedure for different polysaccharides fractions
